# Trends in Cumulative Disenrollment in the Medicare Advantage Program, 2011-2020

**DOI:** 10.1001/jamahealthforum.2023.2717

**Published:** 2023-08-25

**Authors:** David J. Meyers, Andrew M. Ryan, Amal N. Trivedi

**Affiliations:** 1 Department of Health Services, Policy, and Practice, Brown University School of Public Health, Providence, Rhode Island

## Abstract

**Importance:**

The Medicare Advantage (MA) program is rapidly growing. While previous work has found that beneficiaries with substantial health needs disenroll from plans at higher rates, the long-term frequency of disenrollment is not well understood.

**Objective:**

To compare cumulative disenrollment trends in the MA program by beneficiary and plan characteristics.

**Design, Setting, and Participants:**

This retrospective, serial cross-sectional study included beneficiaries with any MA enrollment from January 1, 2011, to December 31, 2020. Data analysis took place from September 2022 to March 2023.

**Exposures:**

Beneficiary characteristics, including race and ethnicity, length of Medicare enrollment, dual eligibility, and comorbidity burden, and contract characteristics, including vertical integration status, premium, and MA star rating.

**Main Outcomes and Measures:**

The main outcome was disenrollment from an MA contract within 5 years. Rates of cumulative disenrollment by beneficiary and contract characteristics were compared. Pearson correlation coefficients were calculated to assess the correlation between a contract’s 1-year disenrollment and the contract’s disenrollment over a longer period.

**Results:**

The sample included 82 377 917 beneficiaries (524 442 225 beneficiary-year observations; 56.7% female; mean [SD] age, 71.9 [10.3] years). After 1 year, 13.2% of nondually enrolled and 15.9% of dually enrolled beneficiaries had left their contract, increasing to 48.3% and 53.4%, respectively, after 5 years. Black enrollees disenrolled at the highest rates among race and ethnicity categories, with 14.8% disenrolling after 1 year and 52.6% disenrolling after 5 years. Contracts had a median disenrollment rate of 9.8% (IQR, 4.5%-19.0%) after 1 year and 56.1% (IQR, 23.1%-79.0%) after 5 years. Contracts rated 5 stars had substantially lower 5-year disenrollment rates (23.0% after 5 years compared with 41.2% for 4- to 4.5-star contracts and 67.2% for 3- to 3.5-star contracts). Disenrollment from a contract after 1 year was not well correlated with disenrollment after 5 years (*r*, 0.46).

**Conclusions and Relevance:**

This cross-sectional study found substantial cumulative rates of disenrollment from MA plans within 5 years between 2011 and 2020, with wide variation in 5-year disenrollment by contract. The findings suggest that evaluating long-term disenrollment rates in MA performance measures may capture different outcomes than single-year disenrollment alone.

## Key Points

**Question:**

What is the frequency of long-term disenrollment from Medicare Advantage (MA) plans, and does disenrollment vary by contract?

**Findings:**

In this cross-sectional study of 82 377 917 individuals with any MA enrollment from 2011 to 2020, 48% of nondually enrolled and 53% of dually enrolled MA beneficiaries had left their contract after 5 years, with substantial variation by plan.

**Meaning:**

This study found high rates of MA disenrollment from 2011 to 2020, which may reduce plans’ incentives to address the longer-term care needs of beneficiaries.

## Teaser

This cross-sectional study examines cumulative trends in beneficiary disenrollment from Medicare Advantage plans by beneficiary and contract characteristics.

## Introduction

In the Medicare Advantage (MA) program, private plans receive capitated payments to cover the needs of enrollees.^1^ These capitated payments in part are intended to incentivize plans to invest in strategies to improve health and reduce spending for beneficiaries.

A key feature of MA is that beneficiaries may choose from a wide variety of plans in their markets and may change their enrollment on an annual basis. Prior work has found that annual disenrollment rates from MA plans are higher for beneficiaries with greater health needs.^2,3,4,5,6,7,8,9,10,11^ However, little is known about disenrollment over a longer term. While disenrollment reflects beneficiaries’ preferences, churn of beneficiaries into and out of contracts reduces plans’ incentives to invest in longer-term interventions to improve population health since beneficiaries may no longer be enrolled in the plan or may even be enrolled with a competing MA insurer. When evaluating the performance of MA plans, the Centers for Medicare & Medicaid Services (CMS) calculates a measure of disenrollment for each contract each year, capturing how many beneficiaries choose to leave their plan from 1 year to the next.^12,13^ However, as currently constructed, this measure does not capture longer-term dynamics in beneficiary enrollment decisions. Using national enrollment data between 2011 and 2020, we evaluated the patterns of and factors associated with disenrollment from MA plans.

## Methods

### Data Sources

This cross-sectional study was determined to be exempt from review, with a waiver of informed consent, by the Brown University institutional review board because data were deidentified and informed consent would not be feasible with a national sample. We followed the Strengthening the Reporting of Observational Studies in Epidemiology (STROBE) reporting guideline. Our primary source of data for this analysis was the Medicare Master Beneficiary Summary File (MBSF) from January 1, 2011, to December 31, 2020. The MBSF includes a record for each Medicare beneficiary each year and information on MA contract and plan enrollment and beneficiary demographics. Prior to 2016, the MBSF did not include MA contract identification numbers, so we linked data at the beneficiary level to the Healthcare Effectiveness Data and Information Set, which includes contract and plan identification numbers from 2011 to 2015.

In addition to the MBSF, we linked to publicly available MA plan characteristic and star rating files. To assess beneficiary care utilization, we linked to the Medicare Provider Analysis and Review file for hospitalization data, the Outcome and Assessment Information Set for home health visits, and the Minimum Data Set (MDS) for nursing home stays. To assess the comorbidity burden of beneficiaries, we additionally linked to Medicare Part D files, and following previous literature,^14^ we calculated Johns Hopkins Ambulatory Care Group (ACG) System groups^15^ based on pharmaceutical prescriptions. The pharmacy-based ACG measures allow for the calculation of beneficiary risk with a reduced opportunity for variations in coding intensity. For the analysis including the ACG measure, we restricted to contracts that included Medicare Part D benefits.

### Study Sample

The primary study sample included all Medicare beneficiaries with any MA enrollment from January 1, 2011, through December 31, 2020. We classified MA enrollment each month of each year using the MBSF. We excluded beneficiaries who were enrolled in Program for All-Inclusive Care for the Elderly, Medical Savings Account, Cost, and employer group contracts as they operate differently and follow different enrollment rules than standard MA contracts.

### Disenrollment

Our primary outcome of interest for this study was beneficiaries’ cumulative disenrollment from their MA contract over time. In our primary analytic approach, we sought to calculate this measure in alignment with how the CMS currently calculates its 1-year disenrollment measure but extending the time frame for a longer period. Following the CMS’s approach, we defined a prevalent population of beneficiaries in each MA contract each year (baseline year). We then evaluated whether each beneficiary was enrolled in the same contract the following year and subsequently for up to 5 years following the baseline year. We then moved on to the next year in the sample to serve as the next baseline year and followed subsequent disenrollment from that point. This resulted in each beneficiary potentially being included in the analysis multiple times.

In our primary version of the outcome, we considered someone to have disenrolled if they were either in a different MA contract or enrolled in traditional Medicare (TM). We created a second version of this outcome to specifically measure whether beneficiaries switched to TM. We did not consider switching plans within the same contract to be a disenrollment as plans frequently combine and alter plan benefit designs and shift enrollees between them.

In alignment with the CMS’s approach, we made several exclusions to this measure. Using the CMS plan crosswalk files, we evaluated whether a contract was consolidated or terminated.^16,17^ If a contract was consolidated into another contract, we did not consider the enrollee to have disenrolled. If a contract was terminated, we censored the evaluation of the cumulative disenrollments at that point. We also censored beneficiaries if they moved to a different county, as that would affect their ability to remain enrolled in the same contract. As such, our measures of cumulative disenrollment and switching to TM can be considered voluntary enrollment changes on behalf of the beneficiary. If a beneficiary died in a subsequent year, we censored our measure at that point and did not include that beneficiary in the calculation of disenrollment for that year.

We also defined disenrollment and the analytic sample using several additional sensitivity specifications. First, we used a similar approach as our primary specification except we randomly selected 1 observation year from each beneficiary so as not to count the same beneficiary multiple times in our analysis. Second, we used a similar approach but included only newly eligible Medicare beneficiaries; however, such a restriction limits external validity, as it generally includes only younger beneficiaries. In our third alternative specification, we conducted a survival analysis by identifying the first year that a beneficiary was enrolled in each contract. We then followed that beneficiary for the subsequent 5 years, considering the failure event to be leaving the original contract. Fourth, we also evaluated disenrollment by the number of prior years of enrollment in a contract that each beneficiary had. Fifth, we tested limiting our disenrollment definitions to changes between parent companies rather than contracts.

### Covariates

In our beneficiary-level analysis, we compared disenrollment by full dual eligibility status as assessed by the MBSF, as dually enrolled individuals have additional opportunities to disenroll within a year. We also compared disenrollment over time by race and ethnicity using the RTI International race code available in the MBSF.^18^ The RTI International code is based on race and ethnicity information taken from Social Security information and updated based on an algorithm to improve the reporting of race and ethnicity and more accurately capture Hispanic ethnicity. The RTI International variable includes categories for American Indian/Alaska Native, Asian, Black, Hispanic, White, and other, with other including beneficiaries who do not fall into the included categories. Race and ethnicity were included in the analysis to investigate whether there is differential enrollment between racial and ethnic groups, potentially due to structural barriers. For a subsample of beneficiaries who had Medicare Part D coverage, we also calculated pharmacy-based ACG codes as a measure of beneficiary comorbidity burden and classified beneficiaries into quintiles of comorbidities. We also calculated counts of hospital, nursing home, and home health stays for each beneficiary.

For contract-level analyses, we assigned to each contract the plan type, premium, and enrollment size based on plan characteristic files. Using the MBSF, we calculated annual contract-level measures of the proportion of beneficiaries who were dually enrolled; we also calculated the percentage of beneficiaries who were Black and the percentage of beneficiaries who were Hispanic, because these are the 2 largest racial and ethnic minority groups enrolled in MA and prior work has found that Black and Hispanic beneficiaries tend to enroll in different contracts.^19^ Using a data set developed in previous work, we also assessed whether contracts were vertically integrated with a health system.^20^

### Statistical Analysis

We first compared disenrollment and switching to TM over time at the beneficiary level and by beneficiary characteristics, such as dual enrollment status, race and ethnicity, comorbidity risk score, and the number of prior years of MA enrollment. All of our comparisons by individual-level characteristics were made at the individual level.

Next, to evaluate cumulative disenrollment as a potential contract-level quality measure, we aggregated the beneficiary-level disenrollment variables to the contract level, resulting in a contract-level percentage of beneficiaries who left the contract within 1 to 5 years. We compared the distribution of these contract-level measures. We then calculated Pearson correlation coefficients to assess how correlated a contract’s 1-year disenrollment (similar to the measure currently used in star rating calculations) was to the contract’s disenrollment over a longer period. We also assessed the correlation between each contract’s disenrollment over time with its publicly reported overall plan rating. The overall plan rating is measured using Consumer Assessment of Healthcare Providers and Systems surveys^21^ and is published publicly at the contract level. We then compared disenrollment over time by different contract characteristics. All of our comparisons using contract characteristics were made using contract-level disenrollment rates.

To calculate 95% CIs for comparing individual and contract characteristics, we used bootstrapping. Given the sample size, we took a 20% random sample and then used 500 replications for the individual-level analysis and 1000 replications for the contract-level analysis. Data analysis took place from September 2022 to March 2023. All analyses were conducted in SAS, version 9.4 (SAS Institute) and Stata, version 17 (StataCorp LLC).

## Results

**Figure 1.**
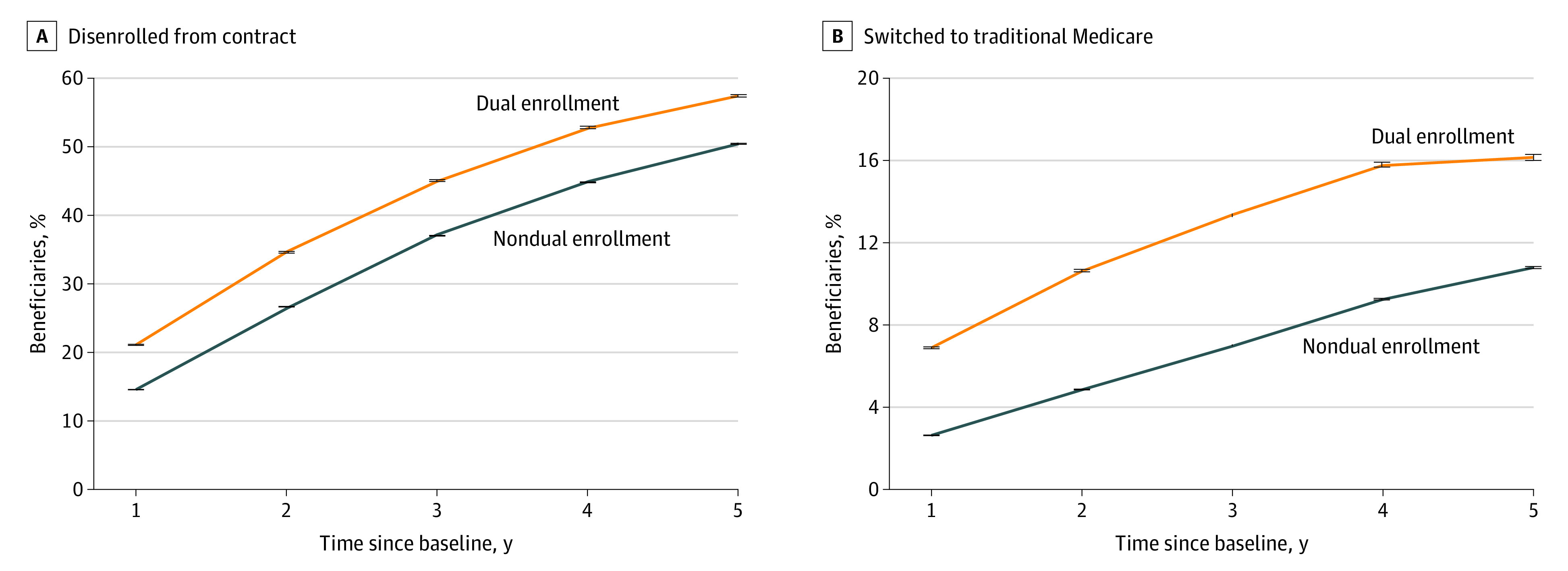
Percentage of Beneficiaries Who Disenrolled or Switched to Traditional Medicare Over Time by Dual Eligibility With Medicaid Disenrollment was defined as a beneficiary voluntarily leaving their contract for either traditional Medicare or another Medicare Advantage contract. Switching was defined as leaving a Medicare Advantage contract and enrolling in traditional Medicare. Error bars represent 95% CIs calculated from bootstrapping.

The primary sample included 524 442 225 beneficiary-year observations representing 82 377 917 unique Medicare beneficiaries from 2011 to 2020. A total of 56.7% were female; 43.3%, male; 0.2%, American Indian/Alaska Native; 3.6%, Asian; 11.9%, Black; 13.9%, Hispanic; 68.6%, White; and 1.8%, other race or ethnicity. Mean (SD) age was 71.9 (10.3) years. Figure 1A presents the proportion of MA beneficiaries who disenrolled 1 through 5 years after baseline, stratified by dual enrollment status. After 1 year, 13.2% of nondually enrolled and 15.9% of dually enrolled beneficiaries had left their contract; after 3 years, 35.0% and 40.3%, respectively, had left their contract; and after 5 years, 48.3% and 53.4%, respectively, had left their contract. In analyses of disenrollment to TM over time, after 1 year, 2.3% of nondually enrolled and 5.8% of dually enrolled beneficiaries had switched to TM, and after 5 years, 8.9% and 13.6%, respectively, had switched to TM (Figure 1B).

**Figure 2.**
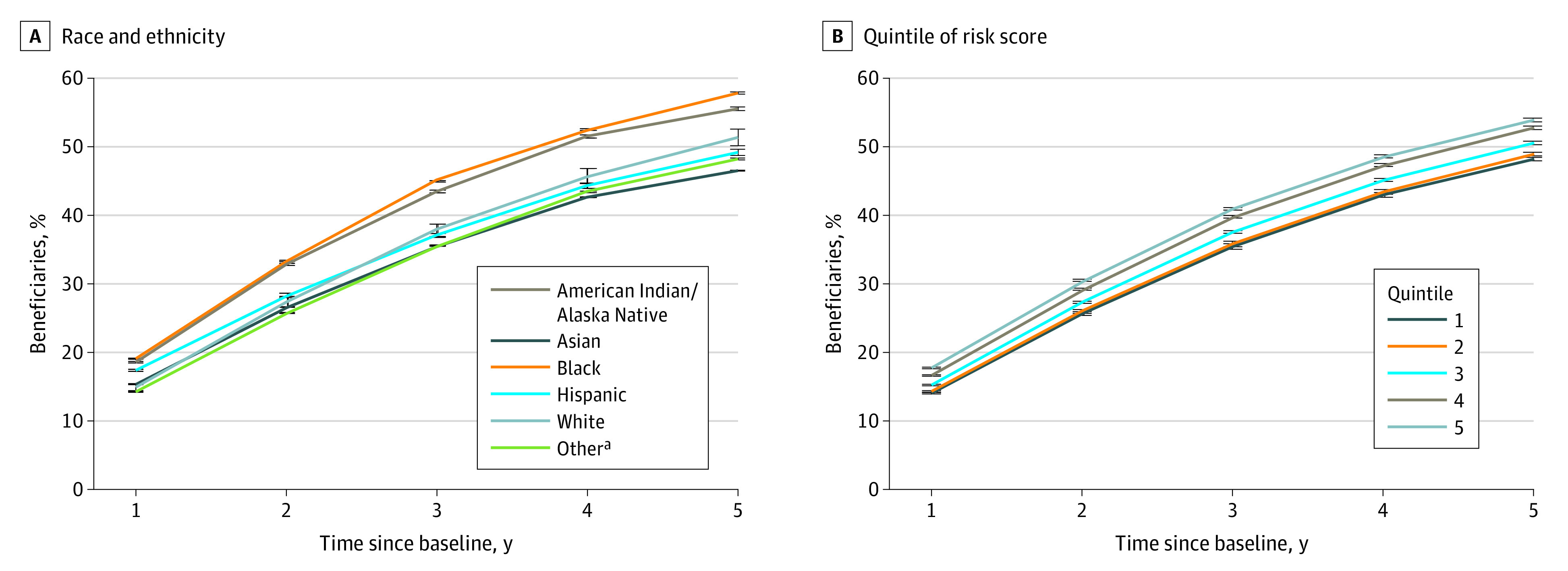
Percentage of Beneficiaries Who Disenrolled From Their Contract by Beneficiary Characteristics Disenrollment was defined as a beneficiary voluntarily leaving their contract for either traditional Medicare or another Medicare Advantage contract. A, Race and ethnicity were included from the Medicare Master Beneficiary Summary File. B, The comorbidity risk score was calculated using Johns Hopkins pharmaceutical-based ambulatory care groups for beneficiaries with Medicare Part D coverage. Error bars represent 95% CIs calculated from bootstrapping. ^a^Includes beneficiaries for whom the Centers for Medicare & Medicaid Services did not have race and ethnicity information and beneficiaries who may identify as having more than 1 race and ethnicity.

Figure 2A shows stratified disenrollment over time by race and ethnicity. We found that Asian and White beneficiaries had the lowest disenrollment over time, with 11.5% of Asian and 12.9% of White enrollees disenrolling after 1 year and 40.7% and 48.1%, respectively, disenrolling after 5 years. Black enrollees disenrolled at the highest rates, with 14.8% disenrolling after 1 year and 52.6% disenrolling after 5 years. We found that beneficiaries with a higher comorbidity burden disenrolled at higher rates (Figure 2B). Compared with a 14.0% 1-year disenrollment rate and a 48.1% 5-year disenrollment rate in quintile 1 of comorbidity, beneficiaries in quintile 5 had a 1-year disenrollment rate of 17.8% and a 5-year disenrollment rate of 54.0%.

Table 1 compares disenrollment over time at the contract level and shows correlations between disenrollment and other variables over time. We found substantial variation in contract-level disenrollment, with a median contract disenrollment rate of 9.8% (IQR, 4.5%-19.0%) after 1 year and 56.1% (IQR, 23.1%-79.0%) after 5 years. While disenrollment after 1 year was generally well correlated with disenrollment after 2 years (*r*, 0.67), the correlation weakened over time, with 1-year disenrollment less correlated with 5-year disenrollment (*r*, 0.46). We also found that increased disenrollment was negatively correlated with overall plan ratings, with a stronger negative correlation over time.

**Table 1.** Variation in Disenrollment Across Contracts and Correlations With Other Measures^a^

Time of disenrollment since baseline, y	Contract distribution, %				*r* ^b^	
	25th Percentile	50th Percentile	75th Percentile	Mean	1-y Disenrollment	Overall plan rating
1	4.5	9.8	19.0	16.7	1	−0.1
2	9.6	20.3	40.3	31.5	0.6	−0.2
3	14.3	31.2	67.2	42.9	0.5	−0.3
4	18.9	44.2	76.0	52.2	0.5	−0.4
5	23.1	56.1	79.0	59.0	0.4	−0.4

^a^ Disenrollment was defined as a beneficiary voluntarily leaving their contract for either traditional Medicare or another Medicare Advantage contract and then aggregated to the contract level. Aggregated disenrollment rates at the Medicare Advantage contract level are shown.

^b^ Pearson correlations between a plan’s disenrollment measure and 1-year disenrollment and beneficiary overall rating of the health plan from Consumer Assessment of Healthcare Providers and Systems performance measurement.

Table 2 compares disenrollment over time by contract characteristics. Preferred provider organizations had a higher mean (SD) 5-year disenrollment rate compared with health maintenance organizations (74.7% [30.5%] vs 54.2% [34.5%]). The contracts with the highest premiums had a lower mean (SD) 5-year disenrollment rate than the $0 premium contracts (56.6% [35.8%] vs 66.3% [32.8%]). Vertically integrated contracts had lower mean (SD) disenrollment rates than nonintegrated contracts after 5 years (40.0% [33.0%] vs 64.3% [33.5%]). Contracts with a greater proportion of Black enrollees also had substantially higher mean (SD) 5-year disenrollment rates vs contracts with a lower proportion of Black enrollees (70.7% [32.7%] vs 53.9% [34.5%]).

**Table 2.** Contract-Level Disenrollment Over Time by Contract Characteristics^a^

Contract type	Contract-years, No.	Disenrollment, mean (SD), %	
		1 y	5 y
Plan type^b^			
PPO	2702	20.7 (23.9)	74.7 (30.5)
HMO	1806	15.7 (18.0)	54.2 (34.5)
Premium, $^b^^,^^c^			
0	498	19.8 (16.9)	66.3 (32.8)
0-30	1492	19.2 (21.0)	66.1 (32.9)
>30	1902	18.1 (24.6)	56.6 (35.8)
Vertical integration status^d^			
Integrated	3273	16.7 (17.6)	64.3 (33.5)
Not integrated	680	12.5 (17.5)	40.0 (33.0)
Dual enrollment, tertile^e^			
1 (0%-8%)	1466	19.0 (24.8)	57.2 (35.4)
2 (9%-33%)	1473	20.3 (22.1)	66.4 (33.8)
3 (>33%)	1247	15.2 (17.4)	61.3 (33.5)
Race and ethnicity, tertile^e^			
American Indian/Alaska Native			
1 (0%-0.05%)	1148	18.7 (23.3)	60.1 (34.6)
2 (0.06%-2.2%)	1639	19.0 (22.7)	62.7 (34.3)
3 (>2.2%)	1399	17.2 (19.9)	62.3 (34.7)
Asian			
1 (0%-1.0%)	1268	18.6 (23.0)	60.9 (35.5)
2 (1.1%-2.0%)	1486	19.4 (23.6)	63.0 (34.7)
3 (>2.0%)	1432	18.3 (19.9)	62.7 (33.2)
Black			
1 (0%-4%)	1438	16.9 (22.6)	53.9 (34.5)
2 (5%-16%)	1492	17.8 (20.6)	63.1 (34.2)
3 (>16%)	1256	20.6 (22.8)	70.7 (32.7)
Hispanic			
1 (0%-2%)	1334	17.8 (23.8)	57.8 (35.5)
2 (3%-10%)	1426	19.3 (23.5)	63.6 (35.1)
3 (>10%)	1426	17.9 (18.2)	64.6 (32.5)
White			
1 (0%-57%)	1333	18.7 (19.1)	69.0 (30.3)
2 (58%-86%)	1434	20.0 (23.9)	65.4 (35.7)
3 (>86%)	1419	16.3 (22.3)	53.0 (34.6)
Contract enrollment^e^			
Small (0-3000)	808	17.9 (18.1)	67.3 (33.5)
Medium (3001-15 000)	1682	18.3 (21.1)	66.4 (34.1)
Large (>15 000)	1688	18.5 (24.2)	53.5 (34.0)

Abbreviations: HMO, health maintenance organization; PPO, preferred provider organization.

^a^ Disenrollment was defined as a beneficiary voluntarily leaving their contract for either traditional Medicare or another Medicare Advantage contract and then aggregated to the contract level.

^b^ Derived from publicly available plan benefit files.

^c^ While premium is a plan-level characteristic, we assigned a contract-level premium as a weighted average of all plan premiums within that contract.

^d^ Integration status was determined using a database of contracts that were vertically integrated with health systems.

^e^ Derived from the Master Beneficiary Summary File and aggregated to the contract-year level.

**Figure 3.**
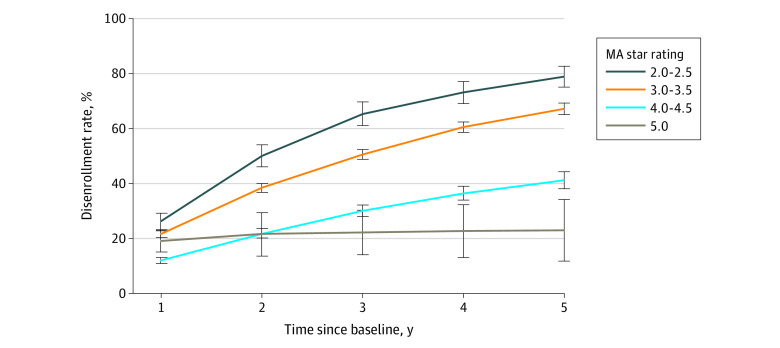
Contract-Level Disenrollment Rates by Overall Medicare Advantage (MA) Star Rating Disenrollment was defined as a beneficiary voluntarily leaving their contract for either traditional Medicare or another MA contract and then aggregated to the contract level. Lines are stratified by publicly reported overall MA star rating. Error bars represent 95% CIs calculated via bootstrapping.

Figure 3 compares disenrollment over time by contract star rating. We found that compared with lower-rated contracts, contracts rated 5 stars had substantially lower 5-year disenrollment rates (23.0% after 5 years compared with 41.2% for 4- to 4.5-star contracts and 67.2% for 3- to 3.5-star contracts).

We calculated the quintile of 1-year disenrollment that each contract was in and assessed whether it was in a higher or lower quintile of performance each subsequent year (eTable 1 in Supplement 1). We found that only 28.4% of contracts were in the same disenrollment quintile after 5 years, with 53.6% in a higher quintile of overall disenrollment.

eFigures 1 and 2 in Supplement 1 present Kaplan-Meier curves stratified by dual eligibility and race and ethnicity from our survival sensitivity analysis. We found similar disenrollment trends over time. We compared disenrollment rates among beneficiaries who were newly enrolled in their contract in the baseline year (eTable 2 in Supplement 1) and restricted our analysis to 1 random observation per beneficiary (eTable 3 in Supplement 1); in both analyses, we found similar results to the primary analysis. We compared trends in these disenrollment rates over time and did not find any consistent trend by year (eTable 4 in Supplement 1). We compared disenrollment by the number of previous years when a beneficiary was enrolled in their MA contract prior to the baseline year and found that the longer a beneficiary was enrolled in their contract, the lower the subsequent disenrollment over time tended to be (eTable 5 in Supplement 1). As shown in eTable 6 in Supplement 1, instead of comparing disenrollment at the contract level, we only considered a disenrollment to be a beneficiary leaving their parent company. When restricting to changes in the parent company, we found lower disenrollment rates overall, but after 5 years, 26.3% of all beneficiaries had still left any MA plan owned by the same company.

## Discussion

Our study has 5 key findings. First, after 5 years, 48.3% of nondually enrolled and 53.4% of dually enrolled MA beneficiaries were no longer enrolled in the same contract. Second, Black beneficiaries and those with greater comorbidity burden had higher 5-year disenrollment rates from their MA contracts. Third, there was substantial variation in 5-year disenrollment across MA contracts, with only modest correlation with contracts’ 1-year disenrollment rates. Fourth, higher-rated contracts and vertically integrated contracts had substantially lower cumulative disenrollment rates. Fifth, most disenrollment over time was to another MA contract rather than the TM program.

This study builds on a prior study that sought to evaluate cumulative disenrollment over time^22^ and expands on it in 3 key ways. First, our study used a longer study period from 2011 to 2020. Second, we did not limit our analysis to new MA beneficiaries, which may expand the external validity as we included a larger set of beneficiaries. Third, we quantified variations in 5-year disenrollment by race and ethnicity, clinical complexity, and contract-level characteristics and evaluated variation in cumulative disenrollment at the contract level.

Our findings have important implications for the MA program. A key feature of MA is that plans are paid on a capitated basis and are held accountable for quality of care and health care spending for the enrolled population. If, however, after 3 years, over one-third of beneficiaries have left their contract and, after 5 years, nearly half of beneficiaries have disenrolled, plans face diminished incentives to invest in longer-term strategies to improve care and outcomes for beneficiaries. Given the high level of disenrollment within 3 to 5 years found in this study, plans may financially benefit by increasing coding intensity in a short period while avoiding interventions to address chronic conditions in which potential benefits may take time to materialize and accrue to competing insurers.

The levels of disenrollment that we measured are larger than in previous studies that only focused on 1-year disenrollment.^2,3,5,7,9,10,11^ On one hand, these levels of disenrollment may be indicative of a healthy MA marketplace, with beneficiaries freely choosing contracts and making different elections if better choices become available. On the other hand, this could also be a sign of unmeasured discontent with MA contracts, as beneficiaries who disenroll may not be captured in performance measurement for their original plans. Black beneficiaries and beneficiaries with greater health needs also disenrolled at higher rates over time, which may be indicative of unmet needs. In future work, it will be important to understand the plans in which beneficiaries are enrolling instead and whether the plans are any better suited to meet member needs.

At the contract level, there was substantial variation in 5-year disenrollment rates, which were only modestly correlated with 1-year disenrollment. This could be a result of 1-year and 5-year disenrollment rates capturing different constructs of enrollee experience. For example, a plan could implement a care management program for beneficiaries that may not have an immediate effect. Regardless of the source of these differences, our findings may support incorporating long-term disenrollment into MA performance measurement to capture these dynamics over time. Otherwise, as currently constructed, plans may have little incentive to address the longer-term needs of beneficiaries.

We found that higher-rated contracts had lower 5-year disenrollment rates than lower-rated contracts, with 5-star–rated contracts having half or less of the disenrollment of other contracts. While some of these differences could be explained by CMS policies around star ratings that encourage members to select higher-rated contracts, the differences could also indicate that these contracts were excelling in meeting member needs. While we found that disenrollment rates were lower when we restricted analyses to beneficiaries who changed their parent companies, our primary analysis of disenrollment from contracts was still the preferred specification, as contracts are the level at which payment is set and quality is assessed.

### Limitations

Our study has several limitations. First, this analysis was associational and could not evaluate causation. Second, while we censored for mortality, to the extent that plans can change the mortality trajectories of their beneficiaries, differential mortality by contract may bias our results. Furthermore, it has been documented that beneficiaries tend to disenroll at higher rates in the last year of life.^23,24^

## Conclusions

In this cross-sectional study, we found that 48.3% of nondually enrolled and 53.4% of dually enrolled MA beneficiaries disenrolled from their contract within 5 years between 2011 and 2020, with wide variation in 5-year disenrollment at the contract level. As the MA program continues to grow, more attention may be needed to ensure that plans are adequately incentivized to take care of patients over time and to ensure that frequent disenrollments do not lead to disruptions in care.

## Notes

Supplement 1. **eTable 1.** Changes in Quintile of Disenrollment by Year **eTable 2.** Disenrollment Analysis Restricted to Beneficiaries Who Are Newly Enrolled in Their Medicare Advantage Contract **eTable 3.** Primary Results Restricted to 1 Observation per Beneficiary **eTable 4.** Trends in 5-Year Disenrollment Over Time **eTable 5.** Disenrollment by Tenure in Medicare Advantage Contract **eTable 6.** Disenrollment From Parent Company **eFigure 1.** Percentage Remaining in Contracts Over Time by Dual Eligibility **eFigure 2.** Percentage Remaining in Contracts Over Time by Race and Ethnicity

Supplement 2.
Data Sharing Statement
