# Supplementary material for: Trends in Cumulative Disenrollment in the Medicare Advantage Program, 2011-2020
Source: JAMA Health Forum. 2023 Aug 25;4(8):e232717. doi: 10.1001/jamahealthforum.2023.2717 (PMC10457718; doi:10.1001/jamahealthforum.2023.2717)
Supplement: Supplement 1. — eTable 1. Changes in Quintile of Disenrollment by Year eTable 2. Disenrollment Analysis Restricted to Beneficiaries Who Are Newly Enrolled in Their Medicare Advantage Contract eTable 3. Primary Results Restricted to 1 Observation per Beneficiary eTable 4. Trends in 5-Year Disenrollment Over Time eTable 5. Disenrollment by Tenure in Medicare Advantage Contract eTable 6. Disenrollment From Parent Company eFigure 1. Percentage Remaining in Contracts Over Time by Dual Eligibility eFigure 2. Percentage Remaining in Contracts Over Time by Race and Ethnicity [file jamahealthforum-e232717-s001.pdf]

Supplementary Online Content

Meyers DJ, Ryan AM, Trivedi AN. Trends in cumulative disenrollment in the Medicare Advantage program, 2011-2020. *JAMA Health Forum*. 2023;4(8):e232717. doi:10.1001/jamahealthforum.2023.2717

eTable 1. Changes in Quintile of Disenrollment by Year

eTable 2. Disenrollment Analysis Restricted to Beneficiaries Who Are Newly Enrolled in Their Medicare Advantage Contract

eTable 3. Primary Results Restricted to 1 Observation per Beneficiary

eTable 4. Trends in 5-Year Disenrollment Over Time

eTable 5. Disenrollment by Tenure in Medicare Advantage Contract

eTable 6. Disenrollment From Parent Company

eFigure 1. Percentage Remaining in Contracts Over Time by Dual Eligibility

eFigure 2. Percentage Remaining in Contracts Over Time by Race and Ethnicity

This supplementary material has been provided by the authors to give readers additional information about their work.

© 2023 Meyers DJ et al. *JAMA Health Forum*.

eTable 1: Changes in quintile of disenrollment by year

Quintile of 1 Year Disenrollment	2 Year Disenrollment	3 Year Disenrollment	4 Year Disenrollment	5 Year Disenrollment
Same Quintile	60.5	43.4	32.3	28.4
Higher Quintile	22.8	36	44	53.6
Lower Quintile	16.6	20.6	23.8	18

Notes: In this table we first categorized MA contracts in quintiles of disenrollment based on the one-year measure. We then compared if the same contract would be in the same, a higher, or a lower quintile of disenrollment in each year of subsequent cumulative disenrollment. Higher quintiles had higher rates of cumulative disenrollment.

© 2023 Meyers DJ et al. *JAMA Health Forum*.

eTable 2: Disenrollment analysis restricted to beneficiaries who are newly enrolled in their Medicare Advantage contract

	Overall	Non Dual	Dual	White	Black	Other	Asian	Hispanic	AN/AI
1 Year Disenrollment	10.6	10.5	11.7	10.1	11.3	9.4	9.1	13.3	11.6
2 Years Disenrollment	20.0	19.6	22.4	19.5	21.5	17.7	16.6	22.4	21.9
3 Years Disenrollment	29.3	28.8	32.2	29.1	32.4	25.9	23.2	30.1	30.3
4 Years Disenrollment	37.2	36.6	40.5	36.6	40.2	33.7	32.2	39.2	40.8
5 Years Disenrollment	44.7	43.9	49	44.1	47.5	40.5	39.4	46.8	48.9

Notes: In this table we restrict our sample to beneficiaries who were newly enrolled in their MA contract in the baseline year, and compare disenrollment by group over subsequent years.

© 2023 Meyers DJ et al. *JAMA Health Forum*.

eTable 3: Primary results restricted to 1 observation per beneficiary

	Overall	Non Dual	Dual	White	Black	Other	Asian	Hispanic	AN/AI
1 Year Disenrollment	14.0	13.9	14.1	13.9	14.2	12.7	11.8	14.5	16.5
2 Years Disenrollment	27.4	26.6	31.2	27.4	29.5	25.5	23.9	26.4	34.1
3 Years Disenrollment	38.1	37.1	42.8	38.4	41.5	35.7	32.1	35	45.4
4 Years Disenrollment	46.3	45.3	51.3	46.4	49.6	43.9	41.9	43.9	55.9
5 Years Disenrollment	53.5	52.4	59.1	53.7	56.3	50	48.9	51.4	63.3

Notes: This table limits the primary results to one observation for each beneficiary. The observation used for each beneficiary was randomly selected from among that beneficiary's person-years across the study period.

© 2023 Meyers DJ et al. *JAMA Health Forum*.

eTable 4: Trends in 5-year disenrollment over time

	1 Year Disenrollment	2 Year Disenrollment	3 Year Disenrollment	4 Year Disenrollment	5 Year Disenrollment
2011	12.4	22	31.3	39.2	47.4
2012	13.6	25.2	35.3	45	50.5
2013	16.1	29.6	49.9	47.3	53
2014	19.2	33.9	42.5	49.5	54
2015	20.3	31.4	41.6	47.5	
2016	15.9	29.3	36.5		
2017	14.3	23.8			
2018	13.9				

Notes: In this table we compare 1 through 5 year disenrollment rates at each different baseline year in the study period. Longer term disenrollment is not availible for later baseline years due to a limited follow-up window beyond the end ot he study data in 2019.

© 2023 Meyers DJ et al. *JAMA Health Forum*.

eTable 5: Disenrollment by tenure in Medicare Advantage Contract

Tenure in Contract	1 Year Disenrollment	2 Year Disenrollment	3 Year Disenrollment	4 Year Disenrollment	5 Year Disenrollment
0	16.1	28.8	39.7	47.6	53.4
1	14.0	27.1	47.5	44.9	48.9
2	13.3	25.3	35.5	41.0	44.7
3	12.4	24.2	31.7	36.7	39.6
4	12.3	21.3	27.9	32.6	
5	9.8	17.7	23.1		
6	8.5	15.3			
7	8.1				

Notes: This table shows disenrollment rates over time by the number of prior years the beneficiary was enrolled in the same contract. 0 represents that the baseline year was the first year the beneficiary was enrolled in the same contract. 1 indicates that the beneficiary was enrolled in the same contract for 1 year prior to the baseline year when comparing disenrollments.

© 2023 Meyers DJ et al. *JAMA Health Forum*.

eTable 6: Disenrollment from parent company

	Overall	Non-Dual	Dual	White	Black	Other	Asian	Hispanic	AN/AI
1 Year Disenrollment	8.2	7.8	9.8	7.3	9.3	7.2	8.4	11.6	7.8
2 Years Disenrollment	14.2	13.6	16.8	12.9	16.3	12.7	14.5	18.8	13.7
3 Years Disenrollment	19.2	18.5	22.3	17.6	22	17.2	19.1	24.6	18.4
4 Years Disenrollment	23.1	22.3	26.7	21.3	25.9	21	22.5	29.1	22.4
5 Years Disenrollment	26.3	25.6	29.8	24.5	29.2	23.7	24.5	32.8	25.3

Notes: In the primary analysis, we considered a beneficiary to have voluntarily disenrolled if they were in a different MA contract in the following year. As the same parent company could own multiple contracts, in this analysis we restricted disenrollment to be if a beneficiary left their original parent company.

© 2023 Meyers DJ et al. *JAMA Health Forum*.

eFigure 1: Percentage remaining in contracts over time by dual eligibility

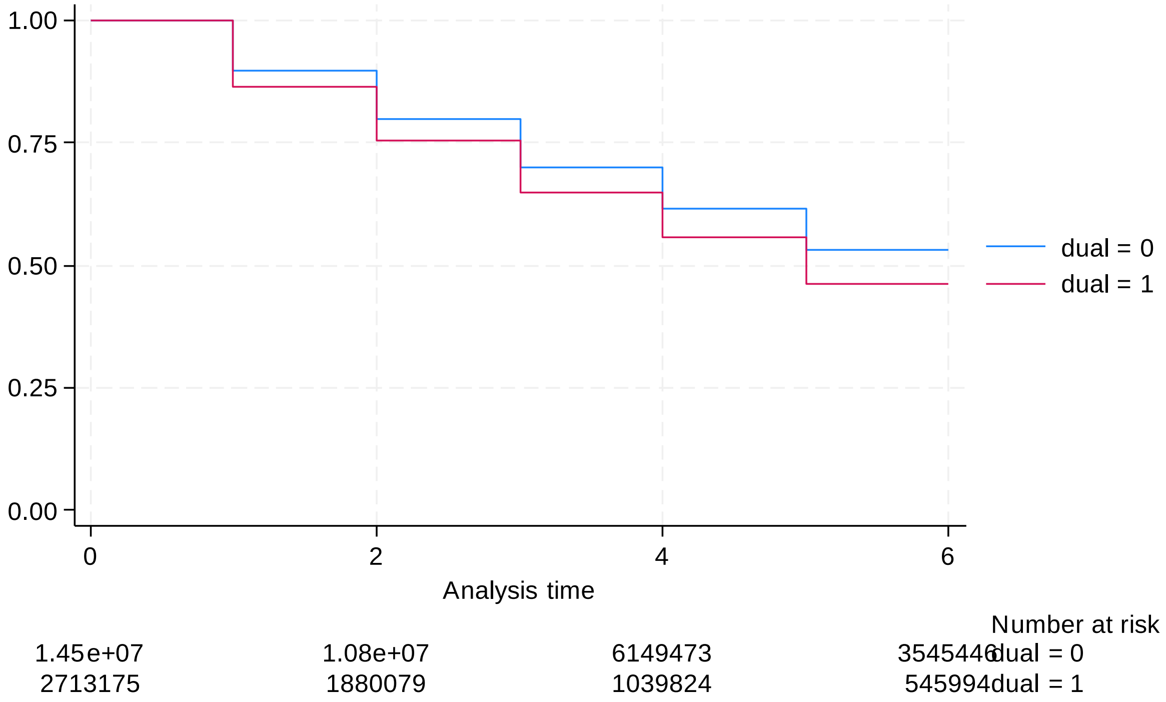

Notes: This figure presents Kaplan-Meier curves starting with a sample of beneficiaries newly enrolled in a given MA contract, and following their continued enrollment in the same contract for up to five years, stratified by dual status.

© 2023 Meyers DJ et al. *JAMA Health Forum*.

eFigure 2: Percentage remaining in contracts over time by race and ethnicity

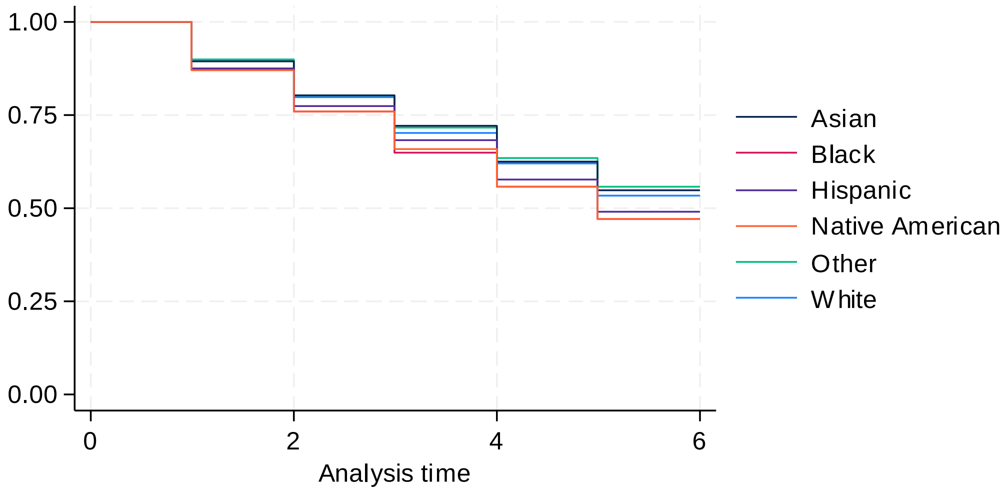

Number at risk

1.23e+07	9040804	5068698	2890858	race2 = white
2009961	1431720	754249	411436	race2 = black
263925	184921	100612	56163	race2 = other/unknown
535911	402306	254973	153638	race2 = asian
2070022	1578993	997350	572351	race2 = hispanic
37891	25719	13415	6994	race2 = native american/alaka native

Notes: This figure presents Kaplan-Meier curves starting with a sample of beneficiaries newly enrolled in a given MA contract, and following their continued enrollment in the same contract for up to five years, stratified by race/ethnicity Other includes beneficiaries for whom race/ethnicity is missing or does not fit into the other categories.

© 2023 Meyers DJ et al. *JAMA Health Forum*.
