# Supplementary material for: Trends in Cumulative Disenrollment in the Medicare Advantage Program, 2011-2020
Source: JAMA Health Forum. 2023 Aug 25;4(8):e232717. doi: 10.1001/jamahealthforum.2023.2717 (PMC10457718; doi:10.1001/jamahealthforum.2023.2717)
Supplement: Supplement 2. — Data Sharing Statement [file jamahealthforum-e232717-s002.pdf]

Data Sharing Statement

Meyers. Trends in Cumulative Disenrollment in the Medicare Advantage Program, 2011-2020. *JAMA Health Forum*. Published August 25, 2023. doi:10.1001/jamahealthforum.2023.2717

Data
Data available: Data are not available for sharing as they are only available under a data use agreement with the Centers for Medicare & Medicaid services
